# Pomegranate Peel Extract as an Inhibitor of SARS-CoV-2 Spike Binding to Human ACE2 Receptor (*in vitro*): A Promising Source of Novel Antiviral Drugs

**DOI:** 10.3389/fchem.2021.638187

**Published:** 2021-04-28

**Authors:** Annalisa Tito, Antonio Colantuono, Luciano Pirone, Emilia Pedone, Daniela Intartaglia, Giuliana Giamundo, Ivan Conte, Paola Vitaglione, Fabio Apone

**Affiliations:** ^1^Arterra Bioscience SPA, Naples, Italy; ^2^Institute of Biostructures and Bioimaging, National Research Council, Naples, Italy; ^3^Telethon Institute of Genetics and Medicine, Pozzuoli, Italy; ^4^Department of Neuroscience, Reproductive and Odontostomatological Sciences, University of Naples Federico II, Naples, Italy; ^5^Department of Biology, University of Naples Federico II, Naples, Italy; ^6^Department of Agricultural Science, University of Naples Federico II, Portici, Italy; ^7^Vitalab Srl, Naples, Italy

**Keywords:** pomegranate peels, SARS-CoV-2, ACE2, COVID-19, polyphenols, pomegranate (*Punica granatum* L.) peel extracts, polyphenols

## Abstract

Plant extracts are rich in bioactive compounds, such as polyphenols, sesquiterpenes, and triterpenes, which potentially have antiviral activities. As a consequence of the coronavirus disease 2019 pandemic, caused by the severe acute respiratory syndrome coronavirus-2 (SARS-CoV-2) virus, thousands of scientists have been working tirelessly trying to understand the biology of this new virus and the disease pathophysiology, with the main goal of discovering effective preventive treatments and therapeutic agents. Plant-derived secondary metabolites may play key roles in preventing and counteracting the rapid spread of SARS-CoV-2 infections by inhibiting the activity of several viral proteins, in particular those involved in the virus entry into the host cells and its replication. Using *in vitro* approaches, we investigated the role of a pomegranate peel extract (PPE) in attenuating the interaction between the SARS-CoV-2 Spike glycoprotein and the human angiotensin-converting enzyme 2 receptor, and on the activity of the virus 3CL protease. Although further studies will be determinant to assess the efficacy of this extract *in vivo*, our results opened new promising opportunities to employ natural extracts for the development of effective and innovative therapies in the fight against SARS-CoV-2.

## Introduction

Plants synthesize a large variety of secondary metabolites having a wide range of biological activities and vital roles for their development and survival (Isah, 2019). Most of those metabolites serve as the plant's defense chemicals against both biotic stresses (e.g., herbivore insects, parasitic nematodes, and microbial pathogens) and abiotic stress (e.g., low or high temperatures, deficient or excessive water, high salinity, heavy metals, and UV radiations) (Yang et al., 2018). For centuries, humans have used plant extracts for medicinal and beneficial health purposes, even though the active compounds responsible for the extract efficacy were mostly unknown. There are thousands of examples of the use of plant-derived compounds as drugs, nutraceuticals, and cosmetic ingredients (Nasri et al., 2014; Atanasov et al., 2015; Barbulova et al., 2015). The active compounds within plant extracts are mainly secondary metabolites that can be classified into four main categories according to their different chemical properties and structures: terpenoids, polyphenols, nitrogen, and sulfur-containing compounds (Ahmed et al., 2017).

Polyphenols are the largest and the most widely distributed group of bioactive compounds in the plant kingdom. They have a distinctive structural skeleton consisting of one or more aromatic phenyl rings connected to hydroxyl groups and exhibiting a wide spectrum of health-related properties including antioxidant protection, anti-inflammatory, anti-allergic, anti-atherogenic, and anti-cancer (Gorzynik-Debicka et al., 2018; Serreli and Deiana, 2019). Moreover, several studies demonstrated the antiviral potential of some classes of polyphenols against Epstein–Barr virus (Yiu et al., 2010), enterovirus 71 (Zhang et al., 2015), herpes simplex virus (HSV) (Annunziata et al., 2018), influenza virus (Lin et al., 2015), and other viruses causing respiratory tract-related infections (Zang et al., 2011). The mechanisms underpinning the antiviral activity of polyphenols are varied (for a review, see Denaro et al., 2020) and include the inhibition of the virus entry because of their permanent attachment on the virion envelope (Lin et al., 2011) or the inhibition of the enzyme responsible for the virus replication (Nutan et al., 2013). The severe acute respiratory syndrome coronavirus-2 (SARS-CoV-2) is a zoonotic pathogenic virus identified for the first time in December 2019 (Zhu et al., 2020), responsible for one of the most serious pandemics in human history, the coronavirus disease 2019 (COVID-19): so far, the number of COVID-19 cases have amounted to over 60 million people with more than 1.4 million deaths from all over the world (https://covid19.who.iht/). SARS-CoV-2, like other coronaviruses, is an enveloped positive-sense single-stranded RNA virus exposing a highly glycosylated Spike (S) protein on its surface, which facilitates the viral entry into host cells. Entry depends on the binding of the surface unit S1 (a portion of the S protein) to a cellular receptor, facilitating viral attachment to the surface of target cells (Hu et al., 2020). Upon binding of the S protein to the host receptor angiotensin-converting enzyme 2 (ACE2), the virus uses the cellular serine protease TMPRSS2 for the priming of S protein itself (Hoffmann et al., 2020). The transcription of TMPRSS2 is promoted by androgen receptors, which could explain the predominance and the severity of pathological signs in COVID-19-affected men compared with women (Guan et al., 2020; Remuzzi and Remuzzi, 2020), the higher proportion of men's hospitalization (Espinosa et al., 2020) and their higher mortality rates (Onder et al., 2020).

Even though recently, alternative molecular mechanisms were hypothesized to explain the virus's entry into the cells (Pirone et al., 2020; Tresoldi et al., 2020), the binding of SARS-CoV-2 S protein to human ACE2 remains the main route of the virus's access to the cells and more directly related to the subsequent levels of infectivity (Davidson et al., 2020). After the virus's entry, the RNA genome is released into the cytoplasm and translated into two polyproteins using the translational machinery of each host cell. The two polyproteins are cleaved into the virus proteins by the main protease M^pro^ (Anand et al., 2003), also referred to as 3CL^pro^, and the papain-like protease PL^pro^ (Shin et al., 2020), while the RNA gets replicated by its own RNA-dependent RNA polymerase (Ahmad et al., 2020). Once the components are all assembled, matured, and packaged into new viral copies, the viruses can then exit the host cell via exocytosis and continue their infection cycles. Sars-CoV-2 mainly targets the respiratory system, intestine, cardiovascular tissues, brain, and kidneys because these organs have the highest expression of ACE2 (Zhang et al., 2020), resulting in symptoms such as fever, headache, dry cough, and dyspnea (Pascarella et al., 2020). At the moment, there are no generally proven effective therapy for COVID-19 but, thanks to joint efforts of scientific communities, three vaccines are now available worldwide (https://ourworldindata.org/covid-vaccinations). As reviewed by Dube et al. (2020), antivirals can be broadly categorized into two classes: the first includes those targeting viral proteins involved in the viral life cycle or virus structure, and the other those mostly targeting host proteins, which are important for viral infection or the host's immune response.

A large number of plant-derived compounds are under investigation for their potential therapeutic effects against SARS-CoV-2. Many reports based on molecular docking analysis suggested the potential capacity of polyphenols, such as curcumin, kaempferol, catechin, naringenin, quercetin (Khaerunnisa et al., 2020) or hesperidin, rutin, and diosmin (Adem et al., 2020) to inhibit the activity of SARS-CoV-2 main protease, and consequently, the virus replication. One study also suggested that the binding of two polyphenols, punicalagin (PC), and theaflavin, to the S protein, could be exploited as a strategy to inhibit the virus's entry into human cells (Bhatia et al., 2020).

Pomegranate (*Punica granatum L*.) fruits, extensively produced by Mediterranean countries, including Tunisia, Turkey, Egypt, Spain, Morocco, and Italy, are rich in polyphenols, such as ellagitannins (ETs), mainly including α and β isomers of PC, gallic acid (GA), ellagic acid (EA), and its glycosylated derivatives, and anthocyanins (Reddy et al., 2007). Pomegranates are majorly processed by food industries to obtain juices or jams from the arils, while the peels, which constitute around 50% of the fresh fruit weight, are discarded. It has been reported that the peels had a higher content of dietary fiber and total polyphenols, as well as a stronger antioxidant capacity (AC) than the pulp fraction of the fruit itself, thus they could be a valuable source of extracts for cosmetic and nutraceutical applications (Akhtar et al., 2015). Evidence suggests that these compounds may have protective activity against degenerative chronic diseases, such as some types of cancer, type 2 diabetes, atherosclerosis, and cardiovascular diseases (Viuda-Martos et al., 2010; Landete, 2011). Furthermore, several studies on PPEs focused on their antibacterial and antiviral activity (Howell and D'Souza, 2013) as well as on the property to inhibit influenza (Moradi et al., 2019) and herpes virus replication (Houston et al., 2017). These observations indicated that PPEs may be successfully employed as antiviral agents against SARS-CoV2. Therefore, this work aimed to assess the potential of PPEs to counteract SARS-CoV2 infection. We found that a hydroalcoholic extract obtained from pomegranate peels and its main constituents were able to inhibit the binding between SARS-CoV-2 S glycoprotein and ACE2 *in vitro*, suggesting a potential of the extract in the prevention of SARS-CoV-2 entry into host cells. Moreover, PPE compounds inhibited the virus 3CL protease, indicating a potential use of the extract as a natural remedy to enhance protection against SARS-CoV-2.

## Materials and Methods

### Preparation of PPE

Dried pomegranate peels were provided by Giovomel–Azienda Biologica (Aiello del Sabato, Italy), an Italian company that produces pomegranate juice. The preparation of the PPE was performed by adding 700 mL of a solution ethanol/water (70/30, v/v) to 150 g of dried peels, at 4°C, according to Malviya et al., 2014. The mixture was homogenized 3 min at 1,500 rpm and 2 min at 3,000 rpm using a Grindomix GM 300 knife mill (Retsch GmbH, Haan, Germany). The resulting suspension was left under stirring at 150 rpm for 2 h at 25°C, avoiding light exposure. The suspension was then centrifuged at 6,300 rpm for 10 min at 4°C. The supernatant was filtered through a filter paper (FILTER-LAB, qualitative filter paper, Barcelona, Spain) and concentrated under vacuum in a rotary evaporator (IKA RV8, IKA-Werke GmbH & Co, Staufen, Germany) set to 25°C. Finally, the pH of the concentrated extract was adjusted to 7.0 with 10N NaOH and then freeze-dried until obtaining a fine powder.

### High-Resolution Mass Spectrometry Analysis of PPE

Liquid chromatography–mass spectrometry data were acquired on an Accela U-HPLC system coupled to an Exactive Orbitrap mass spectrometer (Thermo Fisher Scientific, San Jose, CA, USA) equipped with a heated electrospray interface. The chromatographic separation was carried out according to Colantuono et al. (2017). Briefly, we used a Gemini C18-110Å column, 150 × 2.0 mm, 5 μm (Phenomenex, Torrance, CA, USA) heated to 30°C and the mobile phases consisted of 0.1% formic acid water (A) and 0.1% formic acid acetonitrile (B) with a flow rate of 200 μL/min. The dry extracts were dissolved in methanol/water (50:50, v/v) and 10 μL were injected into the column. MS data acquisition was performed in negative ionization modes, in the mass range of m/z 100–1,300. The resolving power was set to 50,000 full width at half-maximum (FWHM, m/z 200) resulting in a scan time of 1 s. The automatic gain control was used in balanced mode (1 × 106 ions); maximum injection time was 100 ms. The interface parameters were the following: spray voltage 3,500 kV, capillary voltage 50 V, capillary temperature 275°C, sheath gas 30 arbitrary units, and auxiliary gas 15 arbitrary units.

Calibration curves were constructed in the linearity ranges of 1 to 50 μg/mL for PC and 0.1 to 5 μg/mL for EA and GA. Metabolite identification was performed using exact mass values up to the fifth decimal digit with mass tolerance ±5 ppm. Table 1 reports the polyphenols identified in PPE and individual molecular formula, retention time, theoretical mass, experimental mass, and error. The amount of each compound in the extract was determined using PC, EA, and GA as reference standards for ETs, EA derivatives (EAs), and GA, respectively. Punicalin (α, β isomers), granatin B, Causarinin, Galloyl-HHDP-hexoside, pedunculagin I (bis-HHDP-hex), and pedunculagin II (Digalloyl-HHDP-hex) were expressed as equivalents of PC. EA hexoside, EA pentoside, EA deoxyhexoside were expressed as equivalents of EA. GA was quantified with the correspondent standard. Total polyphenols were calculated as the sum of all the compounds retrieved.

**Table 1:** High-resolution mass spectrometry identification of the compounds in pomegranate peels extract (PPE) achieved by Orbitrap MS.

**Compound**	**Molecular formula**	**Theoretical**	**experimental**	**Mass accuracy (ppm)**	**Retention time (min)**
		**[M-H]^**−**^*m/z***			
Punicalin	C_34_H_22_O_22_	781.053	781.05389	1.14	6.3–6.7
Punicalagin	C_48_H_28_O_30_	1083.05926	1083.05994	0.63	7.6–7.9
Pedunculagin I (bis-HHDP-hex)	C_34_H_24_O_22_	783.06865	783.06915	0.64	6.8–7.2–7.8–8.3
Pedunculagin II (digalloyl-HHDP-hex)	C_34_H_26_O_22_	785.0843	785.08502	0.92	8.03–8.6–9.1–9.4
Lagerstannin B	C_41_H_26_O_27_	949.05887			
Causarinin	C_41_H_28_O_26_	935.0796	935.08118	1.69	8.2
Galloyl-HHDP-hexoside	C_27_H_22_O_18_	633.07334	633.0741	1.2	8.6
Granatin B	C_41_H_28_O_27_	951.07452	951.07556	1.09	9.2
Ellagic acid hexoside	C_20_H_16_O_13_	463.05181	463.05225	0.95	8.7
Ellagic acid dihexoside	C_26_H_26_O_18_	625.10464			
Ellagic acid pentoside	C_19_H_14_O_12_	433.04125	433.04135	0.23	9.6
Ellagic acid deoxyhexoside	C_20_H_16_O_12_	447.0569	447.05701	0.25	9.7
Ellagic acid	C_14_H_6_O_8_	300.99899	300.99915	0.53	10.4
Gallic acid	C_7_H_6_O_5_	169.01425	169.01378	−2.78	6.4

### Antioxidant Activity of PPE

The AC of PPE was measured by using the ABTS assay as reported by Re et al. (1999). Briefly, a stable stock solution of ABTS·+ was produced by reacting a 7 mmol/L aqueous solution of ABTS with 2.45 mmol/L potassium persulfate (final concentration) and allowing the mixture to stand in the dark at 4°C for 16 h before use. The ABTS·+ solution was diluted with ethanol to an absorbance of 0.700 ± 0.05 at 734 nm. Freeze-dried PPE was appropriately diluted in water and 0.1 mL of reconstituted extract was added to 1 mL of ABTS·+ solution. The mixture was allowed to stand at room temperature for 2.5 min before the absorbance was recorded at 734 nm by using the multiplate reader Victor Nivo (Perkin Elmer, Woodbridge, ON, Canada). Results were expressed as μmol Trolox equivalents (TE)/g of powder.

### SARS-CoV-2 Spike Receptor-Binding Domain (Spike RBD)/ACE2 Binding Inhibitor Assay

The inhibition of the Spike-ACE2 interaction was measured using the SARS-CoV2 Inhibitor Screening Assay kit (Adipogen Corporation, San Diego, CA, USA; Cat. N° AG-44B-0007-KI01). According to the manufacturer's instructions, 100 μL per well of Spike RBD, produced as a recombinant protein in human cells (1 μg/ml), was used to coat a 96-well plate for 16 h at 4°C. The plate was then treated with the blocking buffer (2% albumine bovine serum (BSA) in phosphate buffered saline (PBS)) for 2 h at room temperature, washed in wash buffer (0.1% Tween^®^ 20 in PBS), and incubated with the PPE or compounds for 1 h at 37°C in the Inhibitor Mix Solution (IMS), composed of 0.2% BSA and 0.05% Tween^®^ 20 in PBS, containing biotin-conjugated-ACE2 (0.5 μg/mL). ACE2 was prepared as a recombinant protein in human cells. After incubation, HRP labeled-streptavidin diluted (1:200 dilution) was added to each well and incubated for 1 h at room temperature. The reaction was developed by adding 100 μL of tetramethylbenzidine (Neogen, Lansing, MI, USA) for 5 min at RT and measured at 450 nm by the microplate reader Victor Nivo (Perkin Elmer, Woodbridge, ON, Canada).

### Microscale Thermophoresis (MST)

MST experiments were performed on a Monolith NT 115 system (Nano Temper Technologies, Munchen, Germany) and designed to evaluate the ability of the PPE to bind ACE2, S protein, and RBD (10108-H08H, 40589-V08B, 40592-V08B from Sino Biological, Wayne, PA, USA). The proteins used in the study were: ACE2 (NP_068576.1) (Met1-Ser740), Spike FL (YP_009724390.1) (Val16-Pro1296), and RBD Spike (YP_009724390.1) (Arg319-Phe541); all three produced as recombinant in baculovirus-insect cells and carrying a polyhistidine tag at the C-terminus. Each protein (10 μM) was labeled with NT-647-NHS reactive dye (30 μM) (NanoTemper Technologies, GmbH, München, Germany), which reacts efficiently with the primary amines of the proteins to form a stable dye protein conjugate. PPE was used in the concentration range of 65–1.92 × 10–3 μM in the experiment with ACE2, 32.5–9.92 × 10–4 μM with Spike and 3.25–9.92 × 10–5 μM with RBD Spike, respectively, preparing 16-point serial dilution (1:2) in PBS supplemented with tween 0.05%. These values corresponded to the quantity of PC, the most abundant extract polyphenol, as determined by chemical analysis. The MST was carried out using 100% LED and 20% IR-laser power at 37°C. The ligand in the experiments with Spike FL and RBD induced quenching of fluorescence and an SDS denaturation test (SD test) was performed to confirm the specificity of the interaction. An equation implemented by the software MO-S002 MO Affinity Analysis (Nano Temper Technologies, Munchen, Germany), provided by the manufacturer was used for fitting the normalized fluorescence values at different concentrations of the ligands (Mercurio et al., 2018; Bellia et al., 2019).

### Lentivirus Infection

Human kidney-2 cells (HK-2) were obtained from American Type Culture Collection (ATCC) and were cultured in Dulbecco's modified Eagle medium (DMEM) (EuroClone, Milano Italy) supplemented with 5% (v/v) FBS, 1% insulin-transferrin-sodium selenite media supplement (ITS) (Sigma-Aldrich-Merck KGaA, Darmstadt, Germany) and 1% penicillin-streptomycin. The cells were maintained at 37°C, 5% CO_2_ in a humidified incubator according to the guidelines provided by the vendors, plated in 96-well plates (CellCarrier-96 ultra with lid, Perkin Elmer, Woodbridge, ON, Canada), at a density of 5 × 10^3^ per well in 100 μL culture medium. After 24 h, the cells were incubated with either 0.04 mg/mL of PPE extract or water for 4 h. The cells were then infected with SARS-CoV-2 Spike-pseudotyped lentivirus (Firefly Luciferase SARS-CoV-2 lentiviral particles, GeneCopoeia, Inc., Rockville, MD, USA) and the control vesicular stomatitis virus G (VSVG) protein pseudotyped lentivirus (HLUC-Lv201 Firefly luciferase + eGFP lentifect, GeneCopoeia, Inc., Rockville, MD, USA) at a concentration of 4.9E + 9 GC/mL and 1.2E + 9 GC/mL, respectively. After 72 h, the cells were fixed in 4% paraformaldehyde and washed three times in PBS. Nuclei were counterstained with 4′,6-diamidino-2-phenylindole, and after washing, the cells were imaged by the Operetta High Content Imaging System (Perkin Elmer, Woodbridge, ON, Canada), using a 20× magnification objective. Acquired images were analyzed by the software Columbus (Perkin Elmer, Woodbridge, ON, Canada), version 2.6.0. Image analysis consisted of identifying and counting viral-infected HK-2 cells based on 488-intensity fluorescence. The infection rate was calculated as the ratio between the number of infected cells and the number of total cells counted per well. The plot shows the percentage of 488-positive cells after pomegranate treatment compared with that in H_2_O-treated cells.

### Gene Expression Analysis on HK-2 Cells

Cells were plated in 24-well plates at a density of 5 × 10^4^ per well in a 500-μL culture medium. After 24 h the cells were incubated with 0.04 mg/mL of PPE for 72 h and then collected for RNA extraction, performed by the GeneElute Mammalian total RNA purification kit (Sigma Aldrich, Merck KGaA, Darmstadt, Germany). The RNA was treated with deoxyribonuclease (DNAse) I (Thermo Fisher Scientific, Dallas, TX, USA) at 37°C for 30 min. Reverse transcription was performed using the RevertAid™ First Strand cDNA Synthesis Kit (Thermo Fisher Scientific, Dallas, TX, USA). Semiquantitative RT-PCR was performed with the Quantum RNA™ kit (Thermo Fisher Scientific, Dallas, TX, USA) containing primers to amplify 18S ribosomal RNA (18S rRNA) along with competimers, that reduced the amplified 18S rRNA product within the range to be used as endogenous standard. The amplification reactions were made using specific oligonucleotides by the Mastercycler™ ProS (Eppendorf, Milan, Italy) with the following general scheme: 2 min at 94°C followed by 35 cycles of 94°C for the 30 s, 50°C for 30 s, and 72°C for 30 s, with a 10 min final extension at 72°C. The PCR products were loaded on 1.5% agarose gel, and the amplification bands were visualized and quantified with the Geliance 200 Imaging system (Perkin Elmer, Woodbridge, ON, Canada). The amplification band corresponding to the analyzed gene was normalized to the amplification band corresponding to the 18S and reported as a percentage of untreated controls set as 100%. The used primer sequences for the amplifications were the following: ACE2 Fw ATGTCACTTTCTGCAGCC; ACE2 Rv GTTGAGCAGTGGCCTTACAT; TMPRSS2 Fw ATTGCCGGCACTTGTGTTCA; TMPRSS2 Rv ACAGTGTGCACCTCAAAGAC.

### 5alpha Reductase Activity

Hair follicle dermal papilla cells (HFDPC) were seeded in a 96-well plate at a density of 8 × 10^3^, after 16 h they were stimulated with testosterone 600 nM and treated with pomegranate extract or finasteride 100 nM for 24 h. Another 96-well plate was coated with 100 ng of dihydrotestosterone (DHT)-conjugated BSA, the day after the plate was washed with PBS +0.05% Tween20 and incubated with a blocking solution containing PBS, Tween20, and 3% of BSA for 1 h. After three washes, the plate was loaded with 50 μL of cell supernatants derived from cell treatments, plus 50 μL of biotin-conjugated anti-DHT antibody (1:1,000 dilution in PBS + BSA 1%). After 2 h, the plate was washed three times, 5 μg/mL of peroxidase streptavidin conjugated was added to the plate and incubated for 1 h at room temperature. After three washes,0.5 mg/mL of OPD in 50 mM citrate buffer +0.012% H_2_O_2_ was added and the absorbance was measured at 490 nm by the microplate reader Victor Nivo (Perkin Elmer, Woodbridge, ON, Canada).

### 3CL Protease Activity Assay

To measure the activity of the viral 3CL protease in the presence of PPE extract we used the Untagged (SARS-CoV-2) Assay kit provided by BPS Bioscience (San Diego, CA, USA), according to the procedure described in the provider's instructions. Briefly, 15 ng of 3CL protease was incubated with the extract at the indicated concentrations or with 500 μM of GC376, used as a positive control. After 30 min of incubation at room temperature, the enzymatic reaction was carried on for 24 h by the addition of 40 μM 3CL protease substrate. The fluorescence was measured by the Victor Nivo Microplate reader (Perkin Elmer Woodbridge, ON, Canada) exciting at 360 nm and detecting at 460 nm.

### Statistical Analysis

All the measures were expressed as means ± standard deviations (SD) of three independent experiments. A paired-samples *t*-test was conducted using Microsoft Excel; a *p*-value lower than 0.05 was considered statistically significant.

## Results and Discussion

### Chemical Characterization of PPE

The concentration of polyphenols in PPE is reported in Table 2. ETs were the most abundant compounds. Specifically, PC represented 38.9% of all the polyphenols detected in the extract, followed by pedunculagin anomers and punicalin anomers representing 16.7 and 13.2% of total polyphenols, respectively. These results were in accordance with previous studies published by Lu et al. (2008) and Fischer et al. (2011). The sum of EAs and GA represented 3.9% of the total polyphenols in PPE.

**Table 2:** Total amount of ETs, EA derivatives, and GA in PPE.

**Compounds**	**PPE (mg/g)**
Punicalagin	182.31 ± 0.75
Punicalin	61.95 ± 2.34
Granatin B	61.04 ± 7.25
Causarinin	20.79 ± 2.52
Galloyl-HHDP-hexoside	45.4 ± 1.53
Lagerstannin B	<LOD
Pedunculagin I	50.25 ± 0.98
Pedunculagin II	28.04 ± 0.42
**Ellagitannins**	449.78 ± 8.31
Ellagic acid	10.71 ± 1.17
Ellagic acid hexoside	3 ± 0.13
Ellagic acid pentoside	1.88 ± 0.09
Ellagic acid deoxyhexoside	1.87 ± 0.11
Ellagic acid dihexoside	<LOD
**Ellagic acid derivatives**	17.45 ± 1.49
Gallic acid	0.98 ± 0.11
Total^*^	468.2 ± 9.69

*The amount of each compound in the extract was determined using PC, EA, and GA as reference standards for ETs, EAs, and GA, respectively. Punicalin, granatin B, causarinin, galloyl-HHDP-hexoside, pedunculagin I (bis-HHDP-hex), pedunculagin II (digalloyl-HHDP-hex) were expressed as equivalents of PC. EA hexoside, EA pentoside, EA deoxyhexoside were expressed as equivalents of EA. GA was quantified with the correspondent standard. Total polyphenols were calculated as sum of all the compounds retrieved. The values are expressed as mg/g of dry powder (mean values ± standard deviation). EA, ellagic acid derivatives; ET, ellagitannin; GA, gallic acid; PC, punicalagin; PPE, pomegranate peels extract*.

* *Expressed as sum of mg of punicalagin equiv*.

*+ mg of ellagic acid equiv. + mg of gallic acid equiv.; <LOD, lower than the limit of detection*.

Notably, the AC of PPE measured by the ABTS method was 3,590 μmol TE/g of PPE. Our data showed that 1 g of freeze-dried PPE was obtained by 3.45 g of dried pomegranate peels. According to these data, the correspondent AC calculated for dried pomegranate peels was 1,041 μmol TE/g of dried pomegranate peels, in line with the data showed by Marchi et al. (2015) (872–1,056 μmol TE/g of dried peels) and by Fischer et al. (2011) (1,362 μmol TE/g of dried peels and 2,887 μmol TE/g of dried mesocarp).

### Effect of PPE on Spike/ACE2 Binding

**Figure 1:**
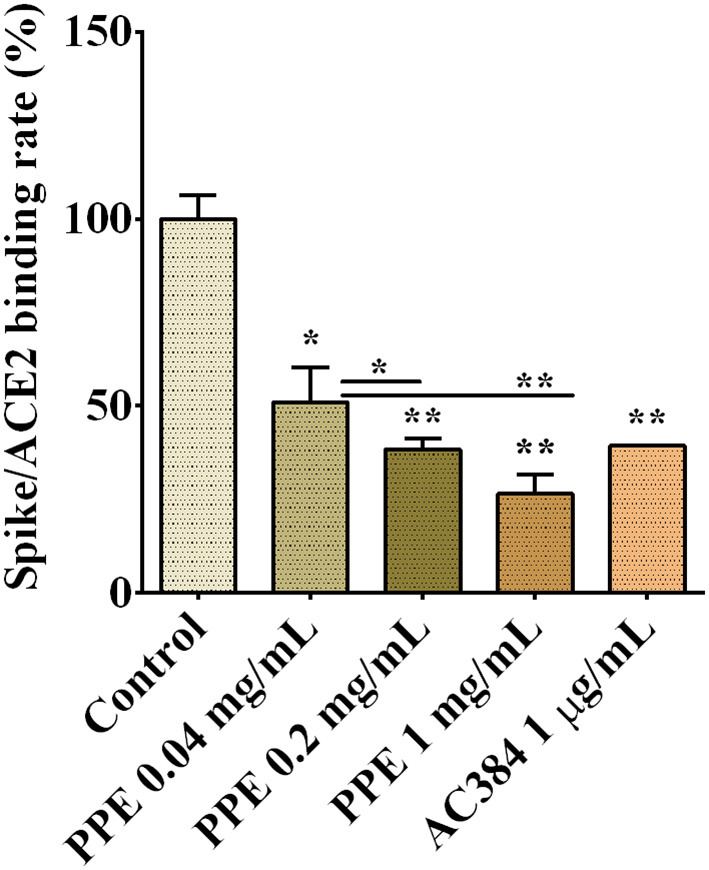
Spike/ACE2 binding in the presence of PPE, used at three concentrations, compared with control and antibody inhibitor AC384. The results are the averages of three independent experiments, expressed as percentages respect to control arbitrarily set as 100%. The error bars represent SDs and the asterisks indicate statistically significant values (^*^p-value is between 0.01 and 0.05; ^**^0.001 and 0.01) according to *T*-test. ACE2, angiotensin-converting enzyme 2; PPE, pomegranate peel extract.

To assess whether PPE had inhibitory activity on Spike/ACE2 binding, we used a SARS-CoV-2 inhibitor screening kit by Adipogen Corporation (San Diego, CA, USA). PPE, used at three concentrations ranging from 0.04 mg/mL to 1 mg/mL, inhibited the interaction between Spike and ACE2 up to 74%, and this effect was dose-dependent (Figure 1 and Supplementary Figure 1). As a positive control, we used AC384, a monoclonal antibody that inhibited the binding between Spike and ACE2 by specifically recognizing ACE2 itself.

To provide insights into which of the PPE polyphenols were relevant for that inhibition, the three most abundant components of PPE, that is PC, EA, and GA were individually tested at the same concentrations as present in 0.04 mg/mL PPE. The results in Table 3 and Supplementary Table 1 showed that PC had the most effect on the binding between Spike and ACE2 by exerting 49% inhibition, followed by EA with 36% inhibition, whereas GA did not have any effect.

**Table 3:** Spike/ACE2 binding (%) in the presence of punicalagin, ellagic acid, and gallic acid, at concentrations corresponding to those present in 0.04 mg/mL of PPE and equal to 7.29, 0.43, and 0.04 μg/mL, respectively.

**Sample**	**Binding (%)**	**SD**	***p*-value**
Spike/ACE2	100	±10	
Spike/ACE2 + PPE	51	±11	0.04
Spike/ACE2 + Punicalagin	36	±4	0.01
Spike/ACE2 + Gallic acid	100	±2	0.5
Spike/ACE2 + Ellagic acid	64	±10	0.03

*The results are the averages of three independent experiments, expressed as percentage respect to control arbitrarily set as 100%. ACE2, angiotensin-converting enzyme 2; PPE, pomegranate peels extract*.

**Figure 2:**
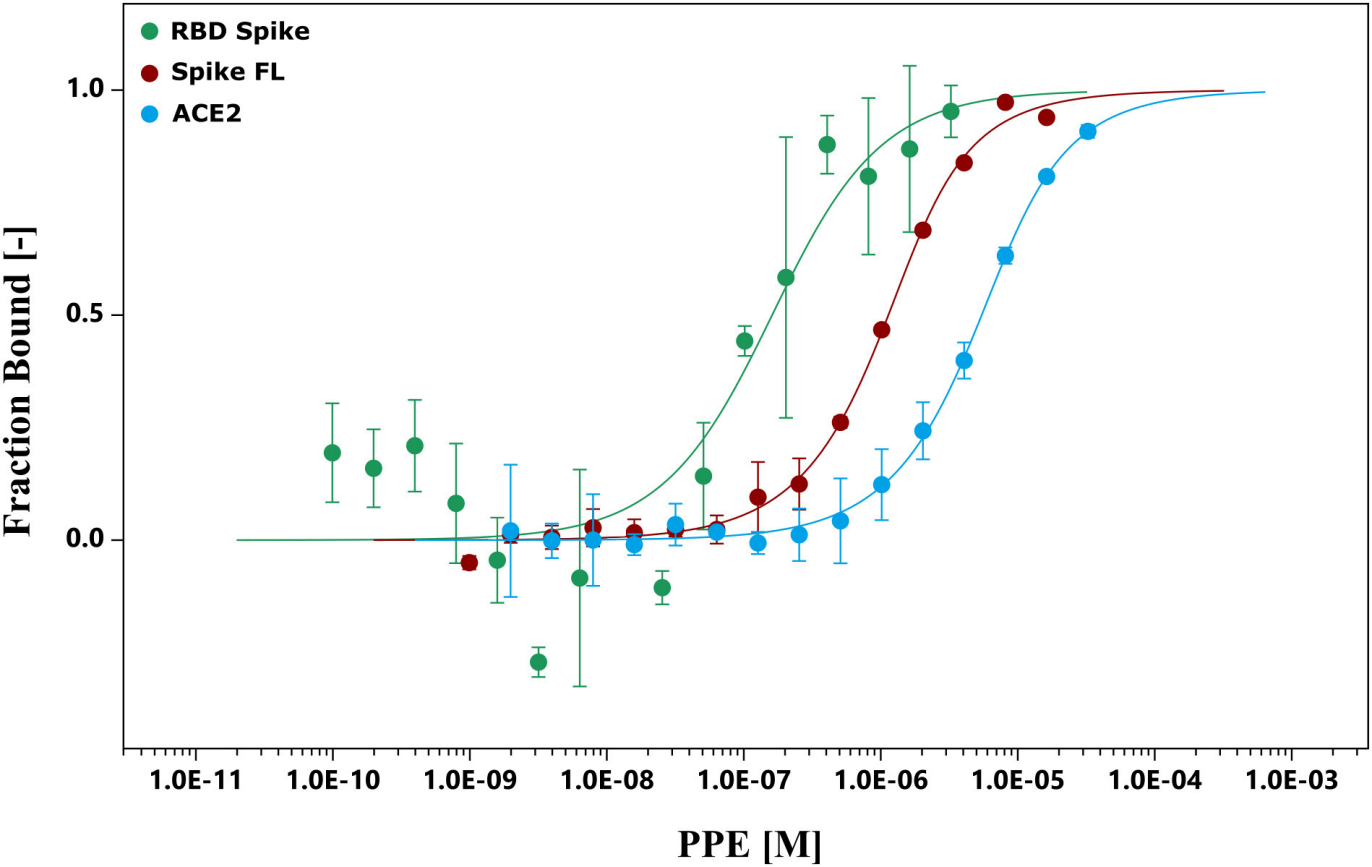
MST. The binding curves were obtained incubating PPE with the RBD Spike, Spike FL protein, and ACE2. ACE2, angiotensin-converting enzyme 2; MST, microscale thermophoresis; PPE, pomegranate peel extract; RBD Spike, Spike receptor-binding domain; Spike FL, Spike full-length.

To further investigate the binding capacity of the pomegranate compounds, the chemical interactions between the extract and Spike, and between the extract and ACE2, were analyzed by MST experiments (Figure 2 and Supplementary Figures 2, 3). The results showed that the PPE bound both the proteins (Figure 2), even though the interaction with Spike was 10-fold as strong as the one with ACE2. Moreover, we supposed that the binding of PPE compounds to Spike was mostly due to a high affinity toward the RBD of the protein, as the binding curve of PPE compounds plus Spike full-length was very similar to that of PPE compounds plus RBD.

**Figure 3:**
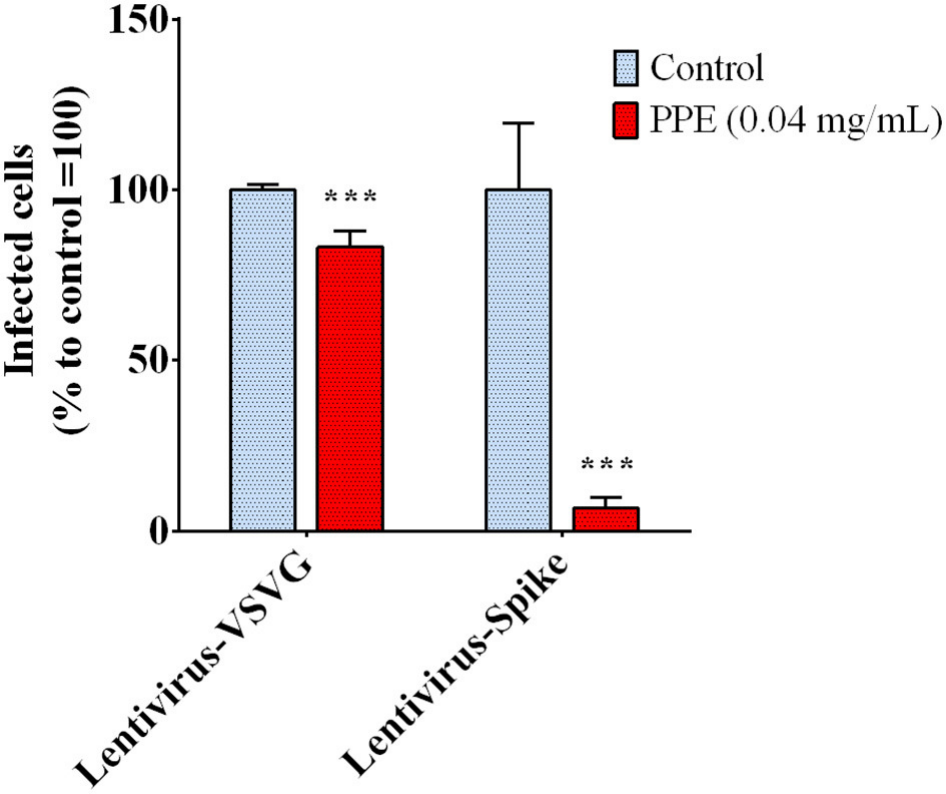
Infection rate of Spike SARS-CoV-2 pseudo-typed lentivirus in HK-2, determined by GFP fluorescence measure. The results are the averages of six independent experiments, expressed as percentages respect to control arbitrarily set as 100%. The error bars represent SDs and the asterisks indicate statistically significant values (^***^p-value is between 0.0001 and 0.001) according to the *T*-test. GFP, green fluorescent protein; HK-2, human kidney-2 cells (HK-2); SARS-CoV-2, severe acute respiratory syndrome coronavirus-2.

The biochemical data prompted us to investigate the capacity of PPE to effectively inhibit the interaction between Spike and ACE2 in a cellular model. To do that, we used a system based on a Spike-carrying Lentivirus, infecting human kidney-2 cells (HK-2), already known to express ACE2 (Koka et al., 2008). As a control, we used a lentivirus that carried the VSVG protein instead of Spike, thus it entered the cells without a specific recognition of any receptor. Both viruses carried the green fluorescent protein (GFP) gene in their RNA genome, which was expressed and easily detected in the cells upon infection. PPE was used at the safe dose of 0.04 mg/mL, as determined by the cytotoxicity MTT assay (data reported in Supplementary Material). As shown in Figure 3, when the cells were infected by the lentivirus carrying the Spike protein in the presence of PPE, the percentage of GFP fluorescent cells (infected cells) was almost significantly abolished after 72 h. In contrast, when the cells were infected by the lentivirus carrying VSVG protein, the percentage of infected cells was reduced only by 18%, suggesting a specific inhibitory effect of PPE toward Spike/ACE2 binding.

**Figure 4:**
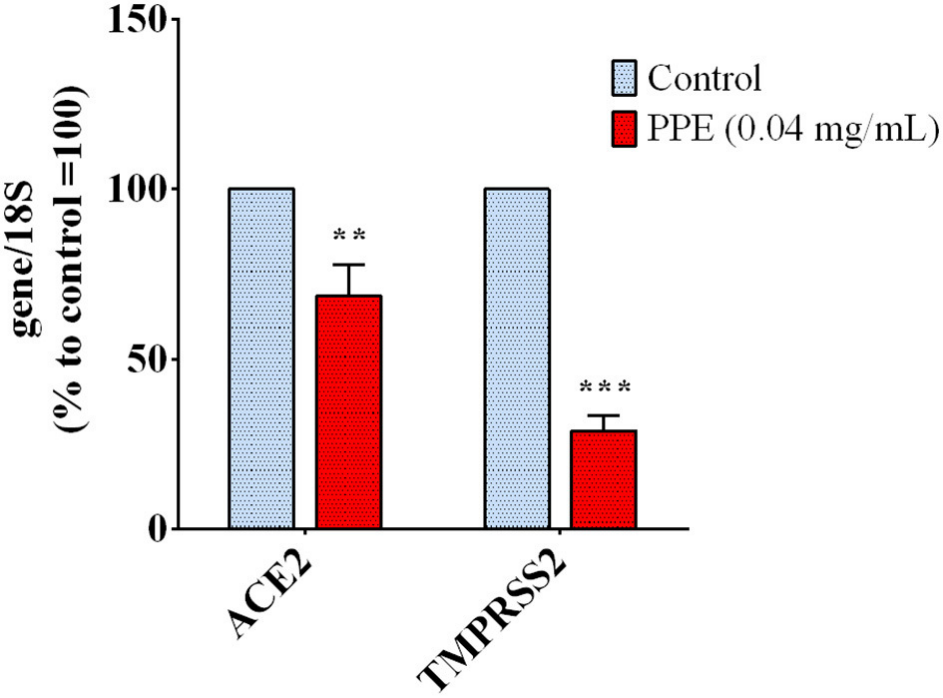
Gene expression analysis in HK-2 cells treated with PPE for 72 h. The results are the averages of three independent RT-PCR experiments. The values are expressed as percentages respect to control arbitrarily set as 100%. The error bars represent standard deviations and the asterisks indicate statistically significant values (^**^p-value is between 0.001 and 0.01; ^***^0.0001 and 0.001) according to *T*-test. HK-2, human kidney-2 cells (HK-2); PPE, pomegranate peel extract; RT-PCR, real-time reverse transcription polymerase chain reaction.

To investigate whether PPE could regulate host genes involved in the virus uptake, we measured the expression level of ACE2 and TMPRSS2 genes in HK-2 cells treated with the extract for 72 h. As reported in Figure 4, the gene expression analysis showed that the treatment of HK-2 cells with the PPE at 0.04 mg/mL reduced the level of ACE2 and TMPRSS2 gene expression by 30 and 70%, respectively. This suggested that PPE, besides Spike/ACE2 binding inhibition, was able to downregulate the expression of two genes responsible for the virus access into the cells.

**Figure 5:**
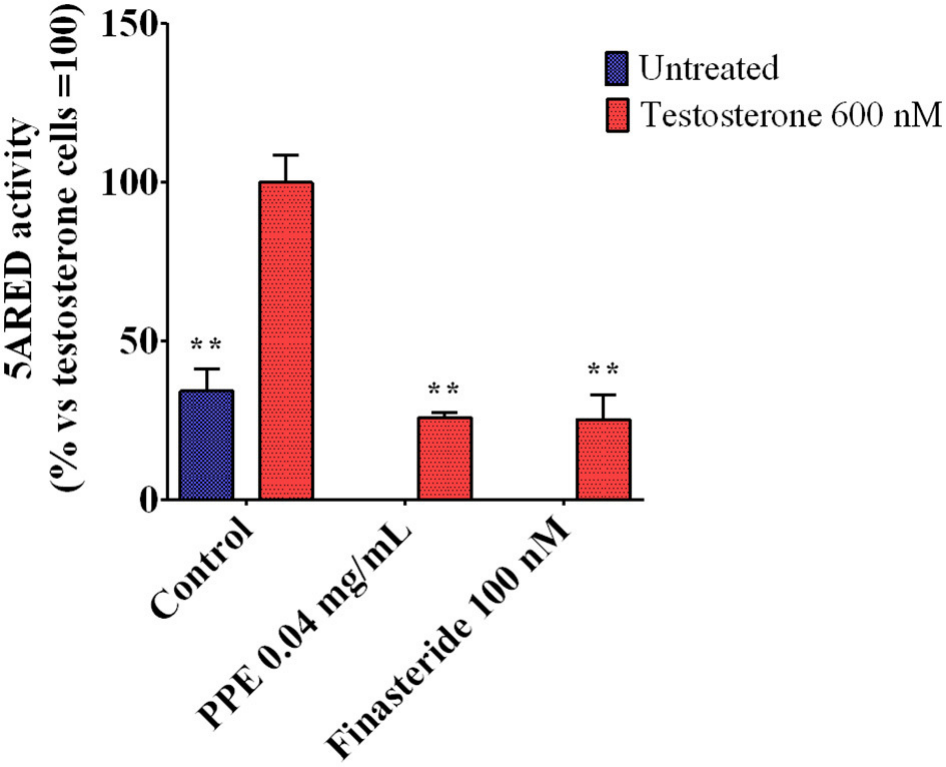
5α-Reductase activity in HFDPC stimulated with testosterone 600 nM and treated with either PPE or finasteride 100 nM. The results are the averages of three independent experiments, expressed as percentages respect to testosterone stimulated cells, arbitrarily set as 100%. The error bars represent SDs and the asterisks indicate statistically significant values (^**^p-value is between 0.001 and 0.01) according to *T*-test. HFDPC, human follicle dermal papilla cells; PPE, pomegranate peel extract.

As the expression of TMPRSS2 was mainly regulated by androgens (Hong et al., 2008; Oyelowo et al., 2019), we analyzed whether PPE inhibited the 5α-reductase activity, the primary enzyme involved in DHT synthesis. As shown in Figure 5, PPE at 0.04mg/mL reduced the activity of the 5α-reductase by 65% in HFDPC, after stimulation by testosterone. This effect was similar to that obtained by finasteride, used as a positive control (Rattanachitthawat et al., 2019).

### Activity of PPE on SarsCov-2 Main Protease

**Figure 6:**
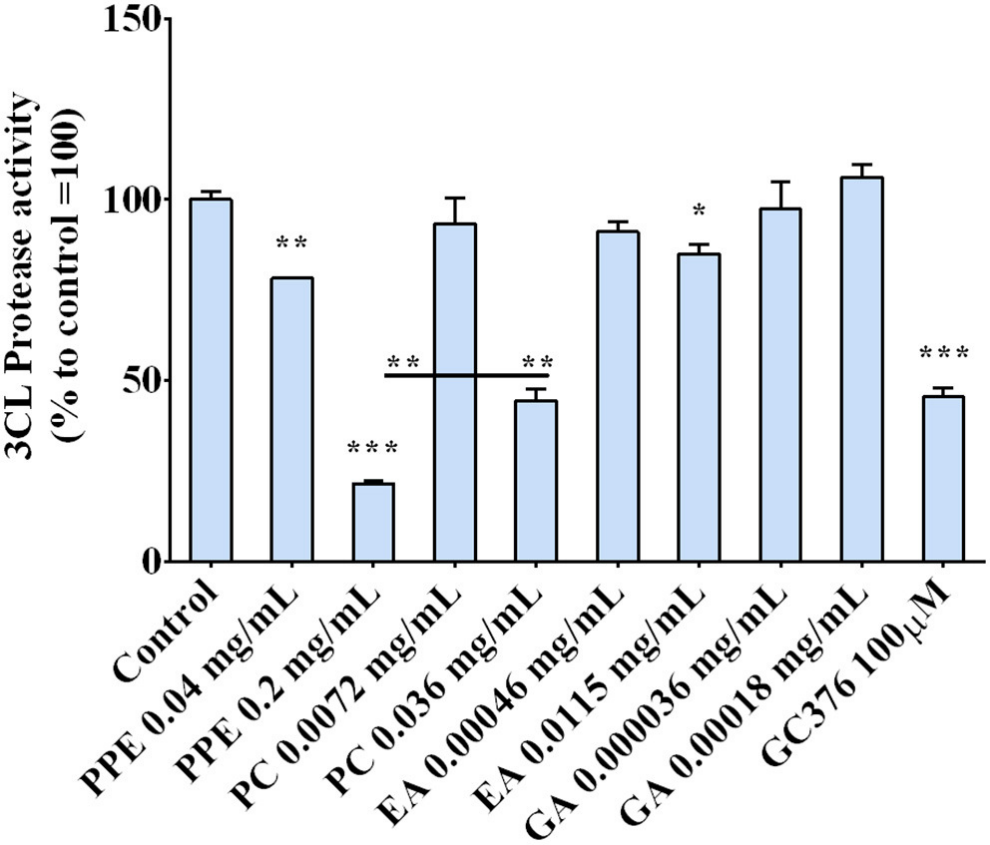
3CL protease activity in the presence of PPE, the main extract compounds (PC, EA, and GA) or GC376 used as positive control. The results are the averages of three independent experiments, expressed as percentages respect to control arbitrarily set as 100%. The error bars represent SDs and the asterisks indicate statistically significantly values (^*^p-value is between 0.01 and 0.05; ^**^0.001 and 0.01; ^***^0.0001 and 0.001) according to *T*-test. PPE, pomegranate peel extract; PC, punicalagin; EA, ellagic acid; GA, gallic acid.

The regulation of the 3CL protease, one of the main proteins involved in the virus replication, by the extract was investigated by incubating the enzyme with PPE and its main components, PC, EA, and GA. The results, reported in Figure 6, indicate that PPE at both concentrations (0.04 and 0.2 mg/ml), inhibited the activity of the 3CL protease up to 80% when used at 0.2 mg/ml. Among the compounds, PC was the most effective in inhibiting the enzymatic activity (by 50%), EA inhibited only by 10%, while GA did not have any effect, suggesting a synergic effect of the PPE polyphenols in inhibiting the protease activity.

## Conclusions

The activity of plants secondary metabolites against SARS-CoV-2 infection and replication has been extensively reviewed in the last months (da Silva Antonio et al., 2020; Sayed et al., 2020; Weng, 2020) and many studies, based on *in silico* approaches, suggested some of them as potential drug candidates for COVID-19 treatment (Majumder and Mandal, 2020; Singh et al., 2020). Both viral structural proteins, like Spike, and non-structural proteins, such as 3CLpro, PLpro, and RdRp, have been proposed as valuable targets for anti-SARS-CoV-2 therapeutic strategies. Through molecular-docking studies, Khalifa et al. (2020) found that some hydrolysable tannins, in particular pedunculagin, tercatain, and castalin, might serve as potential inhibitors of SARS-CoV-2 as they were able to specifically bind the 3CL protease catalytic site.

In parallel studies, Hariprasad et al. (2020) tested the virtual interaction between many plant secondary metabolites and four target proteins involved in COVID-19, the host protease TMPRSS2 and the three virus proteins, Spike, main protease, and RNA-dependent RNA polymerase, and predicted among the class of triterpenoids the most active compounds in blocking the Spike-binding site. Bhatia et al. (2020) also identified PC among dietary polyphenols as a potential inhibitor of Spike and other viral proteases. On the other side, human targets have been taken under consideration as well: ACE2 is certainly the most explored as it turned out to be the main “door lock” that the virus uses to get into the cells. However, ACE2 also has a pivotal role in many physio-pathological processes in human tissues, thus targeting this enzyme needs careful evaluation to ensure that the benefit–risk balance turns favorable (Lacroix et al., 2015; Mostafa-Hedeab, 2020).

In the present study, we found that the polyphenols contained in an ethanolic extract derived from pomegranate peels inhibited the interaction between Spike and ACE2, and reduced the activity of the viral 3CL protease *in vitro*, potentially suggesting the use of the extract as an adjuvant in the treatment against SARS-CoV-2 infections. Data showed that the most effective polyphenols in the extract were PC and EA possibly through a chemical interaction of the hydroxyl and galloyl groups in their molecules with amino acid residues at the active sites of the protein targets of SARS-CoV-2 or human cells, as supported by other studies based on molecular docking analysis (Surucic et al., 2021). The inhibitory effect on Spike/ACE2 binding was confirmed by experiments with a pseudotyped lentivirus, whose entry into the human cells was dependent on Spike protein. Consistent with the *in-vitro* observations, our data showed that lentivirus infection was almost completely abolished by the polyphenol-containing PPE. This inhibition was also associated with the downregulation of the gene expression of both ACE2 and the protease TMPRSS2, the one involved in Spike priming. In particular, in this study 0.04 mg/ml of PPE was used for the *in vitro* experiments in HK-2 cell culture. This concentration appears higher than that used in some reports in which it was indicated that the CC50 of PPE was about 55.6 μg/ml (Moradi et al., 2019). Anyway, in literature, different cytotoxicity data are reported in relation to different cell cultures used (Dana et al., 2015; Mastrogiovanni et al., 2019; Sorrenti et al., 2019; Keta et al., 2020). Moreover, we also provided evidence that PPE was able to inhibit the activity of the 3CL protease up to 80%, suggesting that PPE might have multiple biological roles in reducing the chance of virus to anchor the cells and get internalized.

In conclusion, inhibiting Spike/ACE2 binding still represents one of the most popular strategies to control SARS-CoV-2, and polyphenol-rich extracts represent promising candidates to reduce virus infection and replication thus being proposed as bioactive ingredients in pharmaceutical, nutraceutical, and/or cosmetic formulations. In agreement with our results, a recent report demonstrated that pomegranate juice was effective in reducing the infectious capacity of SARS-CoV-2 and influenza virus in VeroE6 cells suggesting an antiviral activity of both viruses (Frank et al., 2020). The study here presented paves the way for longer and in depth-investigations on the activity of pomegranate peel polyphenols in preventing SARS-CoV-2 infection *in vivo* and it may also promote new ideas on how to reuse an agroindustry byproduct for valuable and healthy applications.

## Data Availability Statement

The datasets presented in this study can be found in online repositories. The names of the repository/repositories and accession number(s) can be found at: MetaboLights, https://www.ebi.ac.uk/metabolights/, MTBLS2309.

## Author Contributions

AT, AC, LP, DI, GG, and PV have performed experimental work. AT, AC, IC, and FA contributed to conception and design of work. AT, AC, EP, and FA contribute to manuscript preparation and revision.

## Conflict of Interest

FA is an Administration Board Member of Vitalab srl. The remaining authors declare that the research was conducted in the absence of any commercial or financial relationships that could be construed as a potential conflict of interest. The reviewer AC declared a shared affiliation with several of the authors, GG, IC, and PV to the handling editor at time of review.

## Abbreviations

PPE: pomegranate peel extract
S-protein: Spike glycoprotein
PC: punicalagin
EA: ellagic acid
GA: gallic acid
EAs: ellagic acid derivatives
ACE2: angiotensin-converting enzyme 2
SARS-CoV-2: severe acute respiratory syndrome coronavirus-2
COVID-19: coronavirus disease 2019.
