# Supplementary material for: Pomegranate Peel Extract as an Inhibitor of SARS-CoV-2 Spike Binding to Human ACE2 Receptor (in vitro): A Promising Source of Novel Antiviral Drugs
Source: Front Chem. 2021 Apr 28;9:638187. doi: 10.3389/fchem.2021.638187 (PMC8114579; doi:10.3389/fchem.2021.638187)

Supplementary material

Supplementary Figure S1: concentration-effect curve of SPIKE-ACE2 binding in presence of PPE.

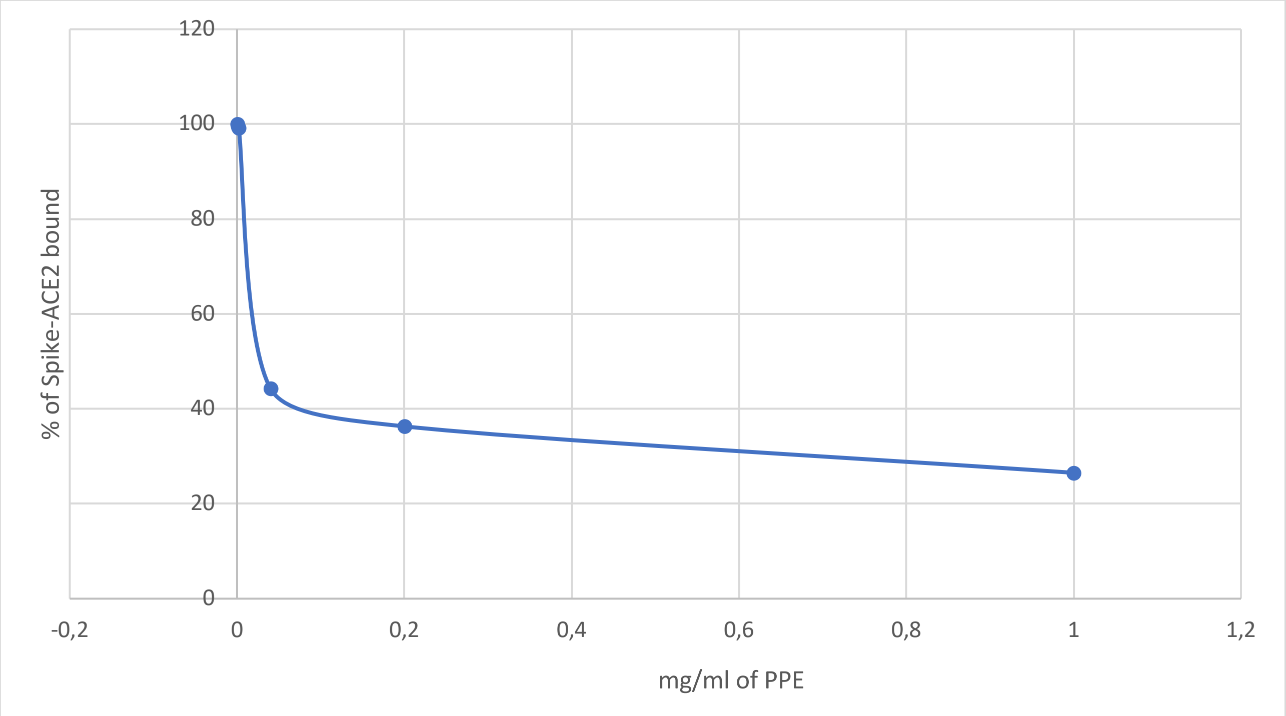

The IC50, calculated using the software provided by the website https://www.aatbio.com/tools/ic50-calculator, was of 0.049mg/ml.

Supplementary Table S1: Spike/Ace2 binding (%) in the presence of punicalagin, ellagic acid and gallic acid, at concentrations corresponding to those present in 0.04mg/ml, 0.008mg/ml and 0.0016. The results are the averages of three independent experiments, expressed as percentage respect to control arbitrarily set as 100%.

	mg/ml of extract		
	0.04	0.008	0.0016
Spike/Ace2	100%	100%	100%
Spike/Ace2 + Punicalagin	36,0	34,1	58,7
Spike/Ace2 + Gallic acid	100,0	100,0	100,0
Spike/Ace2 + Ellagic acid	63,7	59,9	64,4

Supplementary Figure S2: MST traces of titrations of pomegranate extract against Spike (green) and ACE2 (red); F0 and F1 correspond to the fluorescence of unbound state and bound state

respectively.

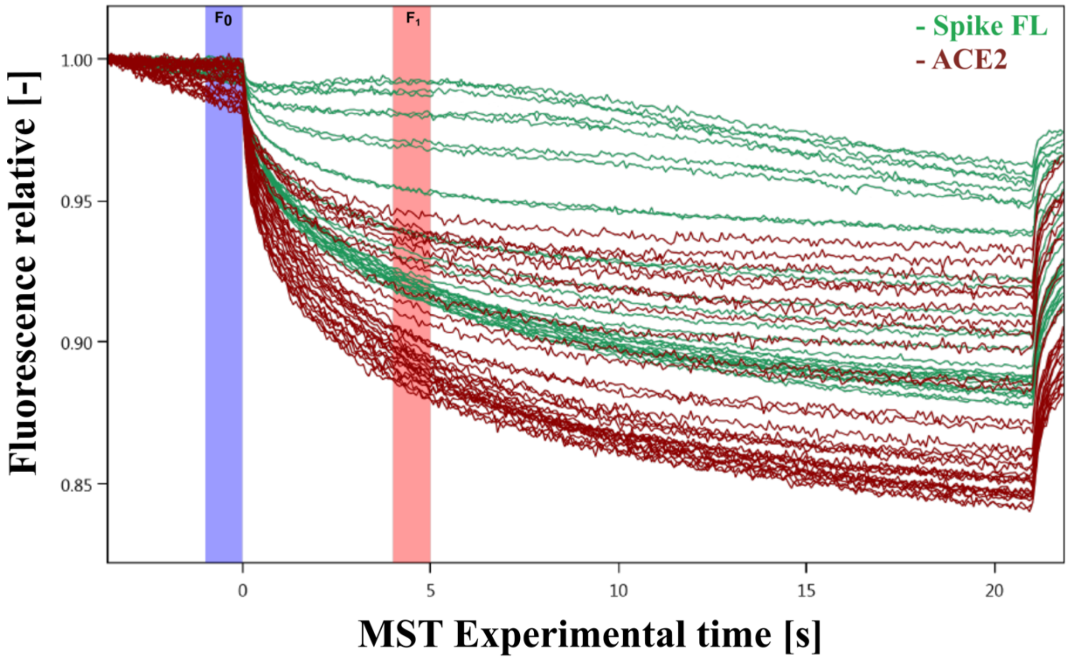

Supplementary Figure S3: MST traces of titrations of pomegranate extract against RBD Spike; F0 and F1 correspond to the fluorescence of unbound state and bound state respectively.

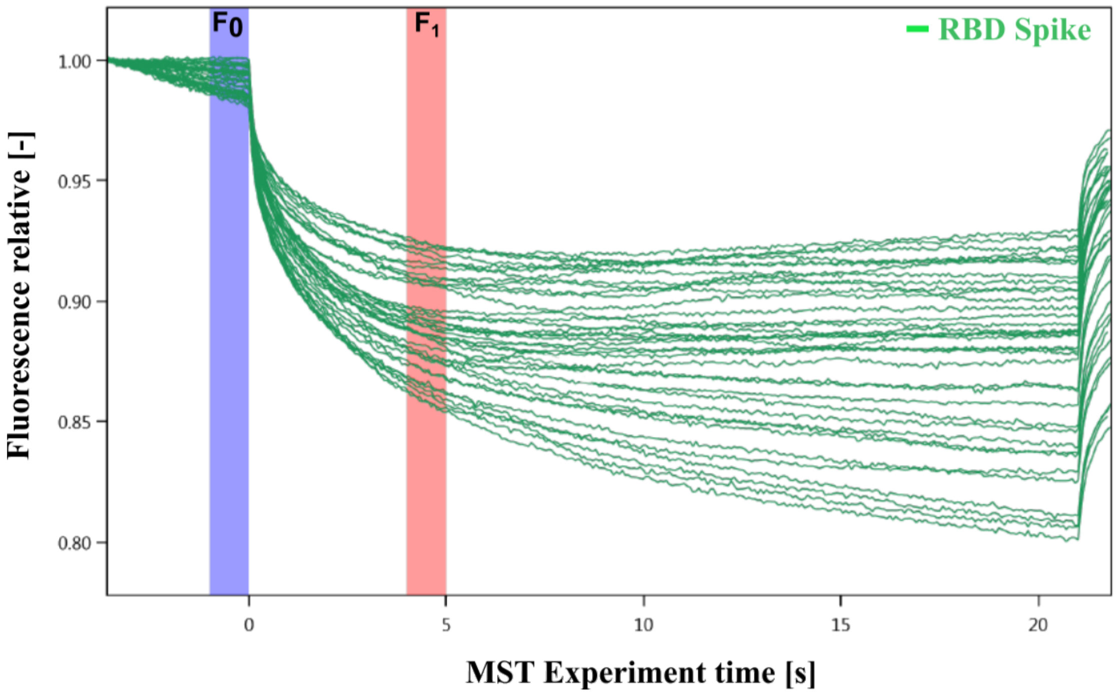

Supplementary Figure 4: MTT assay on HK2 cells

8x10^3 cells were seeded in 96-well plates, grown for 8h and treated for 72h with different concentrations of PPE. After treatments, cells were washed with PBS and incubated with 100 ul/well of "reaction buffer" containing: 10 mM Hepes, 1.3 mM $CaCl_2$, 1mM $MgSO_4$, 5mM glucose and 0.5mg/ml of colorimetric substrate MTT [3-(4,5-dimethylthiazolyl)-2,5-diphenyltetrazolium bromide] in PBS buffer at pH 7.4, according to the method described by Mosmann et al., 1983. After 3h at 37°C and 5% CO_2, cells were solubilized by the addition of a 100 ul of solubilization solution (10% TritonX100, 0.1N HCl in isopropanol), and the plate incubated for 4 hours at room temperature. The number of healthy cells is directly proportional to the level of the formazan product created. The developed color is then quantified at 595 nm by the microplate reader Victor Nivo (Perkin Elmer).

In the figure 4 it was reported the percent of cell vitality in treated cells respect to untreated ones. The CC50, calculated using the software provided on the website https://www.aatbio.com/tools/ic50-calculator, was of 0.24 mg/ml.

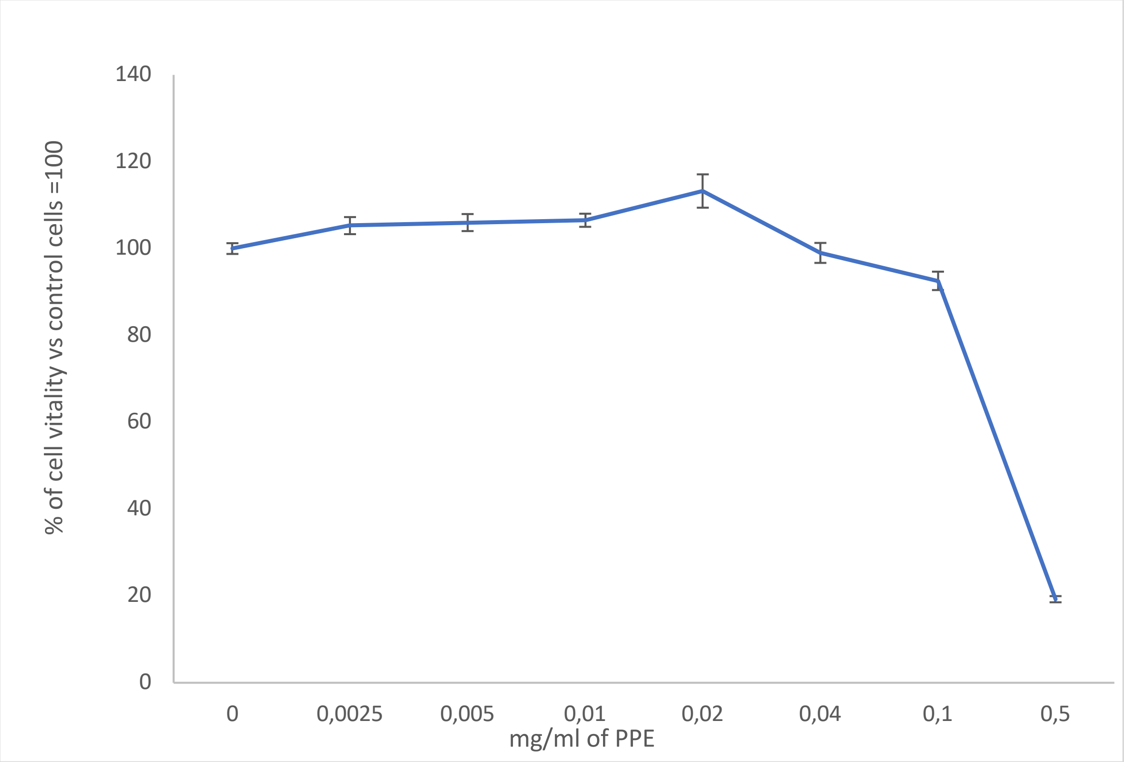

Supplement: Supplementary file 1 [file Data_Sheet_1.pdf]
